# A simple principle concerning the robustness of protein complex activity to changes in gene expression

**DOI:** 10.1186/1752-0509-2-1

**Published:** 2008-01-02

**Authors:** Jennifer I Semple, Tanya Vavouri, Ben Lehner

**Affiliations:** 1 EMBL-CRG Systems Biology Unit, Centre for Genomic Regulation (CRG), UPF, Dr. Aiguader 88, Barcelona 08003, Spain; 2 Institució Catalana de Recerca i Estudis Avançats (ICREA), Centre for Genomic Regulation, UPF, Dr. Aiguader 88, Barcelona 08003, Spain

## Abstract

**Background:**

The functions of a eukaryotic cell are largely performed by multi-subunit protein complexes that act as molecular machines or information processing modules in cellular networks. An important problem in systems biology is to understand how, in general, these molecular machines respond to perturbations.

**Results:**

In yeast, genes that inhibit growth when their expression is reduced are strongly enriched amongst the subunits of multi-subunit protein complexes. This applies to both the core and peripheral subunits of protein complexes, and the subunits of each complex normally have the same loss-of-function phenotypes. In contrast, genes that inhibit growth when their expression is increased are not enriched amongst the core or peripheral subunits of protein complexes, and the behaviour of one subunit of a complex is not predictive for the other subunits with respect to over-expression phenotypes.

**Conclusion:**

We propose the principle that the overall activity of a protein complex is in general robust to an increase, but not to a decrease in the expression of its subunits. This means that whereas phenotypes resulting from a decrease in gene expression can be predicted because they cluster on networks of protein complexes, over-expression phenotypes cannot be predicted in this way. We discuss the implications of these findings for understanding how cells are regulated, how they evolve, and how genetic perturbations connect to disease in humans.

## Background

The proteome of a eukaryotic cell is largely organized as a collection of multi-subunit protein complexes [1-4]. These complexes are defined empirically by the stable association of their subunits during biochemical purification [3,4] and act as molecular machines [5] or information processing modules [6] in cellular networks. For example some of the many integrated complexes required for gene expression include the RNA polymerase complexes, chromatin remodeling complexes, RNA processing complexes such as the spliceosome, exosome and decapping complex, the ribosome, and the proteosome [7].

In this paper we address the question of whether there are any general principles concerning how the activity of protein complexes respond to changes in the expression of their subunits. Available global data in yeast show that reducing the expression of any subunit of a protein complex normally produces the same change in phenotype [8]. However we show here that this is not true for changes in phenotype resulting from increases in the expression of subunits, and this applies to both core and peripheral subunits of complexes. We propose the principle that the overall activity of a protein complex is normally robust to an increase, but not to a decrease in the expression of its subunits. We highlight some of the implications of this principle for understanding the regulation and evolution of biological systems.

## Results

### Genes that reduce fitness when under- but not over-expressed are enriched amongst protein complexes

**Figure 1:**
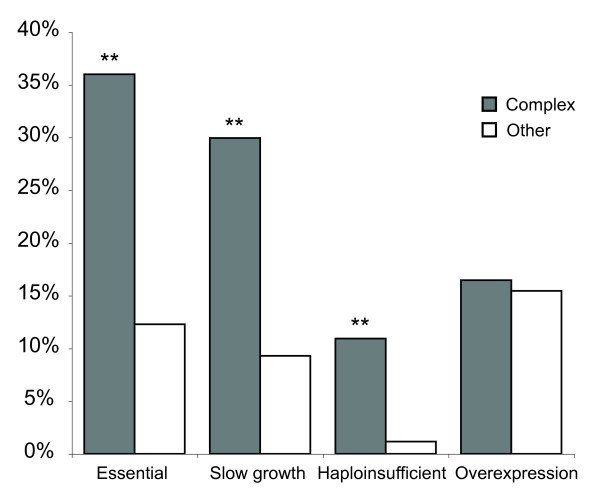
**Genes with under- but not over-expression phenotypes are enriched amongst protein complexes**. Essential genes, genes required for normal growth in rich media and haploinsufficient genes are all enriched amongst the subunits of protein complexes. In contrast genes with over-expression phenotypes are equally represented amongst protein complex subunits and other genes. The graph shows the percentage of genes found in MIPS protein complexes and the percentage of all other genes that have each phenotype. ** Chi square test p < 0.05 for difference between protein complex subunits and all genes.

Most essential functions of the eukaryotic cell are performed by multi-subunit protein complexes. As previously shown [8], genes with essential functions are enriched amongst the subunits of multi-protein complexes (Figure 1). This is also true for haploinsufficient genes (i.e. genes that reduce fitness when their dosage is reduced by half in heterozygotes [9]) and for genes that cause slow growth when they are deleted [10] (Figure 1). Thus inhibiting the expression of a subunit of a protein complex is very likely to disrupt the function of that complex. However genes that slow growth when they are over-expressed [11] (referred to here as genes with over-expression phenotypes) are not enriched amongst the subunits of protein complexes (Figure 1). This lack of enrichment could reflect the fact that many protein complexes are not essential for normal growth and therefore perturbing their function will not result in a visible phenotype. However, we find that genes that reduce fitness when they are over-expressed are also not enriched amongst protein complexes that perform essential functions (Table 1), nor are they enriched amongst the subunits of protein complexes that are essential when deleted (Table 1). Thus in general over-expressing a subunit of an essential protein complex does not normally disturb its function.

**Table 1:** Protein complexes with essential functions are not enriched for subunits with over-expression phenotypes.

	Percentage genes with over-expression phenotype (total number of genes)	P-value
All protein complex subunits	16% (943)	
Complex with no essential subunits	18% (255)	
Complex with at least one essential subunit	15% (688)	0.28^a^
Complex with >= 25% essential subunits	16% (447)	0.56^a^
Complex with >= 50% essential subunits	15% (371)	0.24^a^
Essential subunits of protein complex	14% (342)	0.24^b^

^a ^Significance of difference between the subunits of protein complexes with any, >= 25% or >= 50% essential subunits and the subunits of other complexes (Chi square test, 1 dof).

^b ^Significance of difference between essential and non-essential subunits of protein complexes (Chi square test, 1 dof).

### Genes with under- but not over-expression phenotypes cluster into individual protein complexes

Even if over-expressing a subunit of a protein complex does not in general disrupt the overall activity of the entire complex, it is still possible that a subset of protein complexes may be particularly sensitive to the over-expression of their subunits. To test this we investigated the distribution of genes with under-or over-expression phenotypes amongst complexes. For each phenotype we divided the protein complexes into ten evenly spaced bins according to the fraction of subunits associated with the phenotype. We then compared this distribution of phenotypes to that seen when the subunits are randomized amongst complexes.

**Figure 2:**
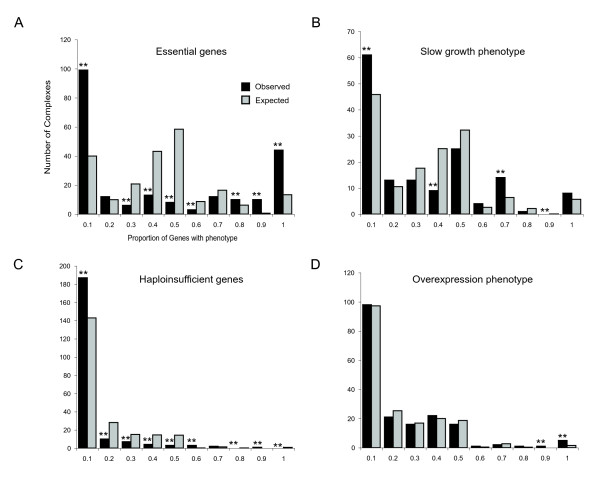
**Genes with under- but not over-expression phenotypes cluster into individual protein complexes**. Genes with essential functions (A), genes required for normal growth in rich media (B), and haploinsufficient genes (C) are arranged amongst protein complexes very differently to the random expectation. In contrast genes with over-expression phenotypes are arranged much more randomly (D). The graphs show the observed number of complexes in each of ten bins defined by the proportion of subunits having each phenotype. These are compared to the expected values (the mean of 100,000 randomisations). ** Bins significantly different from random at a 5% FDR (Benjamini-Hochberg method).

As shown in Figure 2, genes with under-expression phenotypes (essential genes, haploinsufficient genes and genes required for normal growth) cluster into particular protein complexes. For example, 44 complexes have >90% essential subunits compared to 13 expected by chance, and for all phenotypes arising from decreased gene expression there are many more complexes with no genes having that phenotype than expected by chance. In contrast, for genes that reduce fitness when they are over-expressed, only two bins contain more complexes than expected by chance – one complex has 80–90% of tested subunits with an over-expression phenotype (compared to 0.01 expected, p = 0.006) and 5 complexes have >90% of tested subunits with an over-expression phenotype (1.54 expected, p = 0.02). Thus only a few complexes (~3/183) contain more subunits that are toxic when over-expressed than expected by chance. For the vast majority of complexes the distribution of genes with over-expression phenotypes is not different to that expected by chance.

To further confirm this conclusion we asked whether any individual protein complexes contain more subunits with over-expression phenotypes than expected by chance. To do this we randomised the assignment of subunits to protein complexes and for each complex counted the number of times it had the same or more subunits with an over-expression phenotype than seen with the real data. There are 9 complexes with more genes with over-expression phenotypes than in 5% of randomisations, but none of these are significantly enriched for over-expression phenotypes after adjusting for multiple hypothesis testing (see Supplementary table 1 in Additional file 1, Benjamini-Hochberg false discovery rate, FDR = 5%). In contrast, there are 41 complexes with more essential genes than are seen in 5% of randomisations, and 17 of these complexes are still significantly enriched after adjusting for multiple hypothesis testing (see Supplementary table 2 in Additional file 1, FDR = 5%). Indeed the complex most enriched for genes with over-expression phenotypes is the nucleosome complex, and here the over-expression phenotype may be more related to the disruption of the precise temporal regulation of histone expression during the cell cycle [12] rather than disruption of protein complex formation *per se*. Indeed there is an overall enrichment for genes with over-expression phenotypes amongst cell cycle regulated genes (p = 0.037, Fisher's exact test).

Thus we conclude that for protein complexes performing essential functions, inhibiting the expression of any subunit of a protein complex is likely to reduce the overall activity of that complex. In contrast, over-expressing any individual subunit of a protein complex does not normally inhibit the overall activity of that protein complex. This conclusion most likely applies to the vast majority of protein complexes in a eukaryotic cell.

### Neither core nor peripheral subunits of protein complexes are enriched for genes with over-expression phenotypes

**Figure 3:**
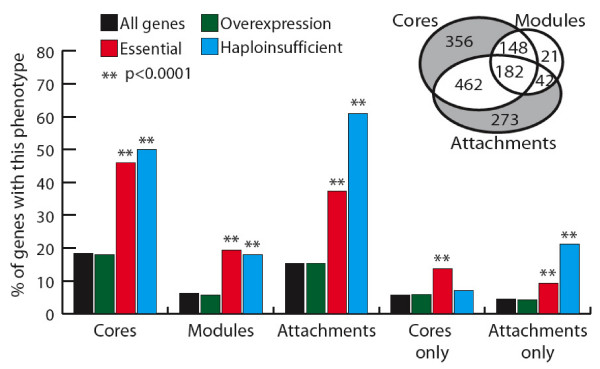
**Genes sensitive to a reduction in expression level, but not to over-expression are enriched amongst both the core and peripheral subunits of protein complexes**. Percentages of genes with essential, overexpression or haploinsufficient phenotypes among different structural components of protein complexes as defined by Gavin *et al. *(2006). The percentages of all genes are shown for comparison. Inset: schematic representation of the overlap between the datasets used. Only 21 genes are found exclusively in modules, so we did not test these as a separate category. ** Fisher's exact test p < 0.0001 for difference between genes with the particular phenotype and all genes without that phenotype.

Previously it has been suggested that subunits that form the structural core of a protein complex might be particularly sensitive to alterations in expression level [13,14]. Therefore we tested whether subunits with under- or over-expression phenotypes are enriched amongst the core or peripheral/isoform-specific subunits of protein complexes. In a genome-wide study of protein complexes identified by tandem affinity purification, Gavin *et al. *identified a total of 491 complexes and classified their subunits as "core" – those present in most complex isoforms, "attachment" – those present only in some isoforms, and "modules" – two or more attachment proteins that tended to occur together in different complexes [3]. As shown in Figure 3, there is no difference between the percentage of genes with over-expression phenotypes in cores, modules, or attachments when compared with yeast genes in general. In contrast, subunits with essential or haploinsufficient phenotypes are significantly enriched among all three types of subunit (p < 0.0001, Fisher's exact test). The same result is seen when only considering genes that fall exclusively within each classification, except that haploinsufficient genes are only enriched amongst attachments (Figure 3).

We conclude that complexes are often sensitive to reduction of a subunit from any part of the complex, and that isoform-specific subunits are particularly sensitive to a partial reduction in the expression of a subunit. These isoform-specific subunits are likely to be regulatory subunits (i.e. limiting the overall activity of a complex) and so may be particularly sensitive to a reduction in expression. In contrast there is no evidence that complexes are sensitive to the over-expression of any particular structural subclass of subunit. Our findings also do not support the previous prediction that the core subunits of protein complexes will be particularly sensitive to over-expression [13,14].

## Discussion

### A simple principle for the robustness of protein complex function and its implications for systems biology

In summary we have shown that in yeast reducing the expression of any individual subunit of a protein complex that performs an essential function under laboratory conditions is likely to disrupt the function of that complex. In contrast increasing the expression of any subunit generally has no effect on the overall activity of a complex. Both of these findings apply equally to core and isoform-specific subunits of protein complexes. Although the over-expression of some complex subunits does result in reduced growth, these phenotypes do not seem related to the disruption of the complex with the possible exception of a very small number of complexes (~3).

**Figure 4:**
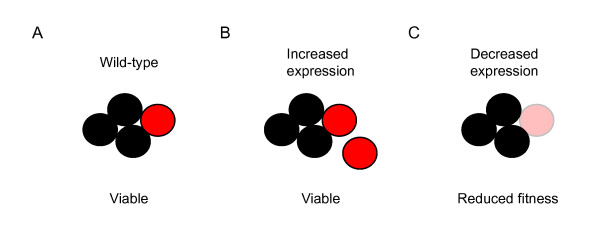
**A simple principle concerning the robustness of protein complex activity**. The results presented here suggest that protein complex activity in eukaryotic cells is in general robust to an increase, but not to a decrease in the expression levels of individual subunits.

Therefore we propose the following principle concerning the robustness of protein complex function to alterations in gene expression (Figure 4): protein complex activity in eukaryotic cells is in general robust to an increase, but not to a decrease in the expression levels of individual subunits. This may reflect either an overall insensitivity of protein complex assembly and activity to the over-expression of subunits or that the cell encodes active mechanisms for degrading subunits produced in excess.

This principle contrasts with previous predictions [13-15] and has several important implications for understanding the design principles and evolution of eukaryotic cells. Here we briefly highlight three implications of the principle: (1) the strategies a cell can use to regulate protein complex function, (2) the trajectories by which eukaryotes can evolve new proteins, and (3) how perturbations of gene expression in human disease can be connected to disease phenotypes.

First, according to the principle, reducing the expression of most subunits of a protein complex will down-regulate the activity of that complex. Therefore there are many alternative strategies available for reducing the activity of a protein complex by altering gene expression. This provides the cell with a very flexible and evolvable framework for regulating protein complex function. In contrast, to up-regulate the activity of a protein complex the cell must coordinately increase the expression levels of all of the subunits, unless the expression of a single subunit is limiting. Thus, in the absence of a limiting subunit [16], up-regulation of complex activity can be most easily achieved by up-regulating a *trans*-acting factor that regulates the expression of all of the subunits.

Second, the insensitivity of protein complex activity to the over-expression of subunits may have facilitated the evolution of novel protein complexes by gene duplication. Most protein complex subunits can probably be duplicated with little phenotypic effect, a situation that would not be true if over-expressing subunits more frequently disrupted the activity of complexes. Indeed such a mechanism of protein complex subunit duplication has been very important in the evolution of new complexes and protein functions [17].

Finally, the principle also has practical implications for understanding the etiology of genetic disease in humans. The results we present here suggest that if a subunit of a protein complex is over-expressed [18] or duplicated [19] in a human disease, then any connection with the disease phenotype is unlikely to be due to an overall reduction in the activity of that complex. Moreover, the fact that genes with over-expression phenotypes do not cluster into protein complexes means that over-expression phenotypes probably cannot be predicted using a comprehensive map of human protein complexes as is possible for loss-of-function phenotypes [20-22]. More sophisticated methods therefore need to be developed to predict the consequences of increases in gene expression levels.

## Methods

### Datasets

769 genes that reduce fitness when they are over-expressed were identified by Sopko *et al*. who tested the phenotypes of 5280 strains each over-expressing a single yeast gene [11]. 1010 essential genes were downloaded from the MIPS database [23]. 184 haploinsufficient genes were identified in a genome-wide screen of heterozygous mutants grown in rich medium [9]. 614 genes required for normal growth in rich media were identified by Giaever *et al.*[10] As a high quality set of protein complexes we used the manually annotated set of MIPS protein complexes (downloaded from MIPS [23] on 14 March 2007, removing one redundantly listed complex, complex 510.190.10.20.10). A second set of systematically identified protein complexes was taken from the data of Gavin *et al.*[3] who classified subunits into cores (1148), modules (393) and attachments (959) of complexes. We used three alternative definitions of an "essential" protein complex – a complex for which at least one, or at least 25% or 50% of subunits have a nonviable deletion phenotype. Cell cycle regulated genes were identified by Spellman *et al.*[12].

### Statistical tests

To compare the distribution of phenotypes amongst protein complexes to that expected by chance we divided the set of protein complexes into ten evenly spaced bins according to the percentage of tested subunits that shared each phenotype. We then randomized the assignment of subunits to protein complexes 100,000 times (but keeping the distribution of complex sizes the same) to calculate the expected frequency of complexes in each bin. To identify bins significantly over- or under-represented for phenotypes we counted the number of times the real enrichments for each bin were seen in the randomizations.

To identify individual complexes significantly enriched for each phenotype we compared the number of subunits of each complex that share a phenotype to the frequencies seen in randomised complexes. To correct for multiple hypothesis testing we used the Benjamini-Hochberg method [24] to identify those complexes enriched at a 5% false-discovery rate (FDR). When testing the association between over-expression phenotypes and protein complex subunits, we only considered complexes for which at least two subunits had been tested for over-expression phenotypes. Hence in this case the total number of complexes considered was 183 rather than 217. The percentages of genes with over-expression phenotypes represent the percentage of tested genes.

## Authors' contributions

JIS, TV and BL analyzed the data and wrote the paper. BL conceived the study. All authors read and approved the manuscript.

## Supplementary Material

Additional file 1 **Supplementary tables 1 and 2**. MIPS protein complexes most enriched for essential genes or genes with over-expression phenotypes (uncorrected p < 0.05). Click here for file

## References

[B1] Aloy P, Bottcher B, Ceulemans H, Leutwein C, Mellwig C, Fischer S, Gavin AC, Bork P, Superti-Furga G, Serrano L, Russell RB (2004). Structure-based assembly of protein complexes in yeast. Science.

[B2] Bork P, Serrano L (2005). Towards cellular systems in 4D. Cell.

[B3] Gavin AC, Aloy P, Grandi P, Krause R, Boesche M, Marzioch M, Rau C, Jensen LJ, Bastuck S, Dumpelfeld B, Edelmann A, Heurtier MA, Hoffman V, Hoefert C, Klein K, Hudak M, Michon AM, Schelder M, Schirle M, Remor M, Rudi T, Hooper S, Bauer A, Bouwmeester T, Casari G, Drewes G, Neubauer G, Rick JM, Kuster B, Bork P, Russell RB, Superti-Furga G (2006). Proteome survey reveals modularity of the yeast cell machinery. Nature.

[B4] Krogan NJ, Cagney G, Yu H, Zhong G, Guo X, Ignatchenko A, Li J, Pu S, Datta N, Tikuisis AP, Punna T, Peregrin-Alvarez JM, Shales M, Zhang X, Davey M, Robinson MD, Paccanaro A, Bray JE, Sheung A, Beattie B, Richards DP, Canadien V, Lalev A, Mena F, Wong P, Starostine A, Canete MM, Vlasblom J, Wu S, Orsi C, Collins SR, Chandran S, Haw R, Rilstone JJ, Gandi K, Thompson NJ, Musso G, St Onge P, Ghanny S, Lam MH, Butland G, Altaf-Ul AM, Kanaya S, Shilatifard A, O'Shea E, Weissman JS, Ingles CJ, Hughes TR, Parkinson J, Gerstein M, Wodak SJ, Emili A, Greenblatt JF (2006). Global landscape of protein complexes in the yeast Saccharomyces cerevisiae. Nature.

[B5] Gunsalus KC, Ge H, Schetter AJ, Goldberg DS, Han JD, Hao T, Berriz GF, Bertin N, Huang J, Chuang LS, Li N, Mani R, Hyman AA, Sonnichsen B, Echeverri CJ, Roth FP, Vidal M, Piano F (2005). Predictive models of molecular machines involved in Caenorhabditis elegans early embryogenesis. Nature.

[B6] Bray D (1995). Protein molecules as computational elements in living cells. Nature.

[B7] Maciag K, Altschuler SJ, Slack MD, Krogan NJ, Emili A, Greenblatt JF, Maniatis T, Wu LF (2006). Systems-level analyses identify extensive coupling among gene expression machines. Mol Syst Biol.

[B8] Hart GT, Lee I, Marcotte ER (2007). A high-accuracy consensus map of yeast protein complexes reveals modular nature of gene essentiality. BMC Bioinformatics.

[B9] Deutschbauer AM, Jaramillo DF, Proctor M, Kumm J, Hillenmeyer ME, Davis RW, Nislow C, Giaever G (2005). Mechanisms of haploinsufficiency revealed by genome-wide profiling in yeast. Genetics.

[B10] Giaever G, Chu AM, Ni L, Connelly C, Riles L, Veronneau S, Dow S, Lucau-Danila A, Anderson K, Andre B, Arkin AP, Astromoff A, El-Bakkoury M, Bangham R, Benito R, Brachat S, Campanaro S, Curtiss M, Davis K, Deutschbauer A, Entian KD, Flaherty P, Foury F, Garfinkel DJ, Gerstein M, Gotte D, Guldener U, Hegemann JH, Hempel S, Herman Z, Jaramillo DF, Kelly DE, Kelly SL, Kotter P, LaBonte D, Lamb DC, Lan N, Liang H, Liao H, Liu L, Luo C, Lussier M, Mao R, Menard P, Ooi SL, Revuelta JL, Roberts CJ, Rose M, Ross-Macdonald P, Scherens B, Schimmack G, Shafer B, Shoemaker DD, Sookhai-Mahadeo S, Storms RK, Strathern JN, Valle G, Voet M, Volckaert G, Wang CY, Ward TR, Wilhelmy J, Winzeler EA, Yang Y, Yen G, Youngman E, Yu K, Bussey H, Boeke JD, Snyder M, Philippsen P, Davis RW, Johnston M (2002). Functional profiling of the Saccharomyces cerevisiae genome. Nature.

[B11] Sopko R, Huang D, Preston N, Chua G, Papp B, Kafadar K, Snyder M, Oliver SG, Cyert M, Hughes TR, Boone C, Andrews B (2006). Mapping pathways and phenotypes by systematic gene overexpression. Mol Cell.

[B12] Spellman PT, Sherlock G, Zhang MQ, Iyer VR, Anders K, Eisen MB, Brown PO, Botstein D, Futcher B (1998). Comprehensive identification of cell cycle-regulated genes of the yeast Saccharomyces cerevisiae by microarray hybridization. Mol Biol Cell.

[B13] Bray D, Lay S (1997). Computer-based analysis of the binding steps in protein complex formation. Proc Natl Acad Sci U S A.

[B14] Veitia RA (2002). Exploring the etiology of haploinsufficiency. Bioessays.

[B15] Papp B, Pal C, Hurst LD (2003). Dosage sensitivity and the evolution of gene families in yeast. Nature.

[B16] de Lichtenberg U, Jensen LJ, Brunak S, Bork P (2005). Dynamic complex formation during the yeast cell cycle. Science.

[B17] Pereira-Leal JB, Levy ED, Kamp C, Teichmann SA (2007). Evolution of protein complexes by duplication of homomeric interactions. Genome Biol.

[B18] Stranger BE, Dermitzakis ET (2006). From DNA to RNA to disease and back: the 'central dogma' of regulatory disease variation. Hum Genomics.

[B19] Beckmann JS, Estivill X, Antonarakis SE (2007). Copy number variants and genetic traits: closer to the resolution of phenotypic to genotypic variability. Nat Rev Genet.

[B20] Lehner B, Fraser AG (2004). A first-draft human protein-interaction map. Genome Biol.

[B21] Franke L, Bakel H, Fokkens L, de Jong ED, Egmont-Petersen M, Wijmenga C (2006). Reconstruction of a functional human gene network, with an application for prioritizing positional candidate genes. Am J Hum Genet.

[B22] Lage K, Karlberg EO, Storling ZM, Olason PI, Pedersen AG, Rigina O, Hinsby AM, Tumer Z, Pociot F, Tommerup N, Moreau Y, Brunak S (2007). A human phenome-interactome network of protein complexes implicated in genetic disorders. Nat Biotechnol.

[B23] Mewes HW, Frishman D, Mayer KF, Munsterkotter M, Noubibou O, Pagel P, Rattei T, Oesterheld M, Ruepp A, Stumpflen V (2006). MIPS: analysis and annotation of proteins from whole genomes in 2005. Nucleic Acids Res.

[B24] Benjamini Y, Hochberg Y (1995). Controlling the false discovery rate: a practical and powerful approach to multiple testing. Journal of the Royal Statistical Society Series B (Methodological).

