# Supplementary material for: A simple principle concerning the robustness of protein complex activity to changes in gene expression
Source: BMC Syst Biol. 2008 Jan 2;2:1. doi: 10.1186/1752-0509-2-1 (PMC2242779; doi:10.1186/1752-0509-2-1)
Supplement: Additional file 1 — Supplementary tables 1 and 2. MIPS protein complexes most enriched for essential genes or genes with over-expression phenotypes (uncorrected p < 0.05). [file 1752-0509-2-1-S1.pdf]

**Supplementary table 1. Protein complexes enriched for subunits with over-expression phenotypes.**

| Complex (MIPS ID) | Description | Subunits (tested) | Fraction essential subunits | P-value[a] | Significant at 5% FDR |
|---|---|---|---|---|---|
| 320 | Nucleosomal protein complex | 8 (6) | 0.83 | 0.00042 | No |
| 480.10 | SPB components | 13 (12) | 0.50 | 0.00591 | No |
| 110 | cAMP-dependent protein kinase | 4 (4) | 0.75 | 0.01266 | No |
| 290.20.20 | Tim17p-complex | 2 (2) | 1.00 | 0.02378 | No |
| 510.190.70 | MBF complex | 2 (2) | 1.00 | 0.02438 | No |
| 190.10 | 1,3-beta-D-glucan synthase | 3 (2) | 1.00 | 0.02439 | No |
| 510.190.60 | SBF complex | 2 (2) | 1.00 | 0.02535 | No |
| 500.20.20 | eEF2 | 2 (2) | 1.00 | 0.02556 | No |
| 260.50.10 | t-SNAREs | 8 (5) | 0.60 | 0.03009 | No |

[a]P-values calculated from 100,000 randomisations (see materials and methods).

**Supplementary table 2. Protein complexes enriched for essential subunits.**

| Complex (MIPS ID) | Description | Subunits | Fraction essential subunits | P-value[a] | Significant at 5% FDR |
|---|---|---|---|---|---|
| 410.30 | Pre-replication complex (pre-RC) | 16 | 1.00 | 0 | Yes |
| 410.35 | Replication complex | 20 | 0.90 | 0.00001 | Yes |
| 510.120 | RNA polymerase III | 13 | 1.00 | 0.00002 | Yes |
| 510.70.20 | TAFIIs | 12 | 0.92 | 0.00017 | Yes |
| 360.10.10 | 20S proteasome | 15 | 0.87 | 0.00019 | Yes |
| 510.100 | TFIIH | 9 | 1.00 | 0.00019 | Yes |
| 440.30.10 | mRNA splicing | 37 | 0.68 | 0.0004 | Yes |
| 130 | Chaperonine containing T-complex TRiC (TCP RING Complex) | 8 | 1.00 | 0.00045 | Yes |
| 410.20 | Replication initiation | 8 | 1.00 | 0.0005 | Yes |

| | | | | | |
|---|---|---|---|---|---|
| | complex | | | | |
| 360.10.20 | 19/22S regulator | 18 | 0.78 | 0.00091 | Yes |
| 440.30.20 | rRNA splicing | 24 | 0.71 | 0.00153 | Yes |
| 500.10.40 | eIF3 | 7 | 1.00 | 0.00164 | Yes |
| 440.14.10 | RNase P | 10 | 0.90 | 0.00173 | Yes |
| 270.20.30 | Dam1 protein complex | 9 | 0.89 | 0.00303 | Yes |
| 440.12.20 | RNase MRP | 9 | 0.89 | 0.00305 | Yes |
| 410.10 | Post-replication complex (Origin recognition complex=ORC ) | 6 | 1.00 | 0.00349 | Yes |
| 510.180.10.30 | NEF3 complex | 9 | 0.89 | 0.00371 | Yes |
| 510.40.10 | RNA polymerase II | 13 | 0.77 | 0.00606 | No |
| 480.10 | SPB components | 13 | 0.77 | 0.00672 | No |
| 410.40.30 | Replication factor C complex | 5 | 1.00 | 0.00881 | No |
| 440.10.10 | pre mRNA3'-end processing factor CFI | 5 | 1.00 | 0.0089 | No |
| 510.150 | TFIIIC | 5 | 1.00 | 0.00913 | No |
| 290.20.10 | Tim22p-complex | 5 | 1.00 | 0.00918 | No |
| 445.10 | SCF-CDC4 complex | 5 | 1.00 | 0.0092 | No |
| 510.10 | RNA polymerase I | 14 | 0.71 | 0.01453 | No |
| 160 | Exocyst complex | 7 | 0.86 | 0.01704 | No |
| 440.12.10 | Exosome complex | 7 | 0.86 | 0.01715 | No |
| 440.10.20 | pre mRNA3'-end processing factor CFII | 4 | 1.00 | 0.0226 | No |
| 410.40.60 | DNA polymerase alpha (I) - primase complex | 4 | 1.00 | 0.02289 | No |
| 270.10.10 | CBF3 protein complex | 4 | 1.00 | 0.0231 | No |
| 510.20 | Core Factor (CF) | 4 | 1.00 | 0.02322 | No |
| 410.33 | GINS complex | 4 | 1.00 | 0.02337 | No |
| 310.10 | NSP1 complex | 4 | 1.00 | 0.02352 | No |
| 270.20.40 | Ndc80 protein complex | 4 | 1.00 | 0.02372 | No |
| 445.30 | SCF-MET30 complex | 4 | 1.00 | 0.0238 | No |
| 60 | Anaphase promoting complex (APC) | 11 | 0.73 | 0.02466 | No |
| 260.90 | Arp2p/Arp3p complex | 6 | 0.83 | 0.03623 | No |
| 475.05 | Sister chromatid cohesion complex | 6 | 0.83 | 0.03662 | No |

| | | | | | |
|---|---|---|---|---|---|
| 260.30.10 | COPI | 8 | 0.75 | 0.04389 | No |
| 260.60 | TRAPP (Transport Protein Particle) complex | 10 | 0.70 | 0.04763 | No |
| 400 | RSC complex (Remodel the structure of chromatin) | 10 | 0.70 | 0.04806 | No |

[a]P-values calculated from 100,000 randomisations (see materials and methods).
